# FUTURE ORIENTATION MEDIATES ASSOCIATIONS BETWEEN ATTITUDE TOWARD OLDER ADULTS AND IMPULSIVITY IN YOUNG ADULTS

**DOI:** 10.1093/geroni/igae098.3272

**Published:** 2024-12-31

**Authors:** Xin Wang, Tianyuan Li

**Affiliations:** The Chinese University of Hong Kong (Shenzhen), ShenZhen, Guangdong, China (People’s Republic); The Chinese University of Hong Kong (Shenzhen), Shenzhen, Guangdong, China (People’s Republic)

## Abstract

Attitude towards older adults (ATOA) is related to young adults’ future expectation, which could also influence their current behaviors. Impulsivity is a risk factor related to a number of health concerns in young adulthood. The current study investigated how ATOA was related to young adults’ impulsive behaviors and explored whether future orientation was a mediator. A total of 371 Chinese young adults were recruited and completed a survey on their demographic characteristic, ATOA, consideration of future consequences (CFC) and impulsiveness. The results showed that ATOA had a significant negative curvilinear relationship with impulsiveness (β = -.04, p =.01). Notably, participants with very positive or very negative ATOA both reported lower impulsiveness level compared with those who were in the middle. Furthermore, CFC mediated this curvilinear relationship (β = -.02, p =.02). There was a significant curvilinear relationship between ATOA and CFC (β =.08, p < 0.001), with both very positive and very negative ATOA related to higher levels of CFC. Higher CFC was further related to lower impulsiveness (β = -.20, p < 0.001). The results suggest that young adults holding very positive or very negative ATOA are both concerned about their future and are more capable of restraining from impulsive behaviors. The inverted U-shaped pattern between ATOA and young adults’ impulsiveness provides a new perspective to understand the role of ATOA in early adulthood. Future studies could further investigate the different motives driving the future orientation for young adults with either positive or negative ATOA.

